
JOURNAL INFORMATION
==============================
NLM Title Abbreviation: ScientificWorldJournal
Journal ID: ScientificWorldJournal
NLM Journal ID: 101131163
Journal ID: TSWJ

The Scientific World Journal

ISSN: 2356-6140
EISSN: 1537-744X
Publisher: Wiley

ARTICLE INFORMATION
==============================
PMCID: PMC5974885
PMID: 12806100
DOI: 10.1100/tsw.2003.27
Article ID: 481519
Article version: 1
Subjects: Editorial

Down from the Ivory Tower: Climate Science Faces the Real World

Hanson Howard *
Los Alamos National Laboratory, MS M708, Los Alamos, NM 87545, USA
*Howard Hanson: hph@lanl.gov
Publication date: 2003
Electronic publication date: 2003 May 5
Volume: 3
First page: 354
Last page: 356
Copyright: Copyright © 2003 Howard Hanson.
Copyright year: 2003
License: This is an open access article distributed under the Creative Commons Attribution License, which permits unrestricted use, distribution, and reproduction in any medium, provided the original work is properly cited.
License URL: https://creativecommons.org/licenses/by/3.0/

==============================
Editorial
*TheScientificWorldJOURNAL* (2003) 3, 354–356
ISSN 1537-744X; DOI 10.1100/tsw.2003.27

TheScientificWorld
www.thescientificworld.com

### Down from the Ivory Tower: Climate Science Faces the Real World

Howard P. Hanson
*Los Alamos National Laboratory, MS M708, Los Alamos, NM 87545*

E-mail: hph@lanl.gov

*Received April 8, 2002; Accepted April 18, 2002; Published May 5, 2003*

---

It would be an overstatement to suggest that the science of Earth's climate system has led a sheltered life. For one thing, it has grown out of a long history of public service (e.g., weather forecasting for the public and private sectors; oceanography and marine meteorology for naval applications). For another, public interest in climatic events — ice ages, nuclear winter, El Niño, sea-level change — is sufficiently high that climate is nearly continuously in the news and has been for years.

However, compared to many other branches of science — biomedicine and nuclear physics come to mind — the community of scientists who investigate the workings of Earth's climate has tended to avoid the spotlight. They did this with fair success until recently, when global warming came along. Now, they are thrust into the glare of public scrutiny due to the controversy over projections of the rise of global temperatures associated with increases of carbon dioxide and other greenhouse gases in the atmosphere.

Earth's climate is an extraordinarily complicated physical, chemical, and biological system. Simulating its behavior is both challenging and fraught with uncertainty due to chaotic processes within the climate system itself and lack of knowledge about future atmospheric composition as well as both known and unknown deficiencies in the computer models used for the simulations. When public scrutiny becomes part of the equation, matters become even more complicated, because explaining the complexities of the climate's behavior to the lay public is a challenge unto itself. Unfortunately, this challenge is not always met successfully.

For example, there is widespread, interchangeable use of the terms "greenhouse effect" and "global warming," an error that introduces only confusion into the ongoing discussion. It is perhaps worthwhile to emphasize the difference between the two here.

What we call, with some imprecision, the **greenhouse effect** is a *climate system process*, in which gases in Earth's atmosphere absorb and re-radiate the heat given off by Earth's surface and by the atmosphere itself. The net effect of this is to insulate the planet and to keep the place livable. Without our atmosphere, Earth's average temperature would be around –18ºC (0ºF) rather than the balmy 15ºC (59ºF) or so that we enjoy today. The greenhouse effect is as real as gravity and, in many respects, more well understood at the quantum level.

**Global warming**, on the other hand, is one possible *result of the net effect of all climate system processes* when the system is perturbed somehow. The perturbation of concern just now,

©2003 with author.

of course, is the input to the atmosphere of carbon dioxide from the combustion of fossil fuels. Because carbon dioxide is a potent greenhouse gas, it is well known that it will further insulate the planet, which, *unless counteracted by other climate system processes*, will result in a temperature increase — global warming. It is these other processes and their net effect that make life interesting, and public, for climate scientists in the early 21$^{st}$ century.

Worth noting is the fact that, just a few years ago, another possible perturbation to the system was of great interest, the injection into the atmosphere of dust and smoke from a global nuclear war. Initially, it was speculated that the net result of this perturbation could be similar to that associated with a large meteor impact, hence the term **nuclear winter**. Further investigation moderated this finding; that is, as research into the issue included more realistic representations of all the relevant climate system processes, it was found that the net effect of all the processes in responding to this possible perturbation was less dramatic than originally thought. Pundits have called this "nuclear autumn."

If the issues surrounding global warming were academic — such as, say, the recent controversy over the proof of Fermat's Last Theorem — they would still receive publicity. But global warming, and especially its mitigation, have real-world economic implications far and wide. Fossil fuels enable our society and are the economic foundation of the developed and developing worlds. As a result, the economic risks of bad climate projections are more than merely significant; they represent insurmountable obstacles to prosperity.

High-quality climate projections are therefore needed, and that is the endeavor in which the climate science community is, and has been for some years, engaged. Despite progress, there remains uncertainty, and this uncertainty generates controversy.

One useful role that venues such as *TheScientificWorldJOURNAL* can play in controversies such as this is to disseminate information and stimulate discussion of the issues involved. It is with this role in mind that we present a review by C.F. Keller[1] of the global warming controversy. Keller discusses several aspects of the global warming problem, aspects that have generated considerable discussion within the climate research community over the past several years. His personal perspective on this discussion provides a window through which TheScientificWorld community can see and understand the difficulties associated with rectifying policy and uncertain science.

Nearly two years ago, the syndicated columnist John Leo, noted for his conservative views on a variety of social issues, wrote about the ongoing debate over global warming. He observed that "...the 'no consensus on warming' crowd now sounds a lot like the tobacco lobby arguing that the link between smoking and lung cancer has not yet been established." Since that time, there have been no new fundamental breakthroughs to shatter the increasingly broad consensus that global warming is happening and that it is being caused by the emission from fossil fuel combustion of carbon dioxide. There continue to be, however, questions about various details of the issues involved, and these details are highly relevant to policymakers in their attempts to develop sound approaches to the problem.

It seems likely that our ultimate strategy to deal with global warming will involve a mixture of various methods that have been suggested. Energy efficiency and developing energy sources on the supply side, and carbon sequestration using natural systems and new technologies downstream will assist society in mitigating future atmospheric $CO_2$ levels. All of these will also provide opportunities for growth across a range of economic sectors. Already, corporate investments in future carbon credits have spawned silvicultural opportunities. Technological approaches to carbon sequestration are moving beyond pure research into the field-test stage. Some of these, especially those involving the oceans, are raising new questions, questions that will require additional research and climate simulation.

Hence, addressing the details that Keller discusses here in *TheScientificWorldJOURNAL* is a task that will not end with a societal commitment to mitigate global warming. How we do the mitigating is potentially as controversial as global warming itself. Indeed, we are seeing the tip of the iceberg of this issue with the current U.S. administration's rejection of the Kyoto Accords and the adoption of its own approaches to the problem. These approaches were the topic of a meeting convened in December 2002 to discuss the administration's Climate Change Science Program Draft Plan[2] (http://www.climatescience.gov/) and to take comments on it. Much of the discussion at that meeting and in the comments on the Draft Plan revolved around issues of uncertainty in climate simulations, as do comments by the National Academy of Sciences panel[3] convened to review the Plan.

Despite this uncertainty and the economic risks associated with policy decisions that affect the fossil-fuel economy, credible voices are urging action. Two recent commentaries illustrate this from separate perspectives.

Kennedy[4] called attention to the dearth of policy decisions related to climate change. He pointed out that the Bush Administration's new program plan does not address a number of issues that are germane to the problem at hand, despite the fact that the "scientific evidence on global warming is now beyond doubt." This is criticism of the top-down management of climate change research, and at a fundamental level it points out the difficulties of formulating public policy in the face of scientific uncertainty.

Pielke and Sarewitz[5] addressed the bottom-up problem, the behavior of the scientific community itself. In rather harsh criticism, they asserted that "...although the current and proposed climate research agenda has little potential to meet the information needs of decisionmakers, it has a significant potential to reinforce a political situation characterized, above all, by continued lack of action." They further suggested that the climate science community's role represents a conflict of interest — promises to reduce uncertainty, after all, serve to continue the funding for the research of the community.

We hope that by publishing reviews of controversial issues such as global warming, *TheScientificWorldJOURNAL* can contribute constructively to the discussion surrounding them and, eventually, to their resolution.

### REFERENCES

1. Keller, C.F. (2003) Global warming: The balance of evidence and its policy implications. *TheScientificWorldJournal*, 3, 357-411.
2. U.S. Climate Change Science Program (CCSP) (2002) Strategic Plan for the Climate Change Science Program (November 11, 2002 Draft), 177 pp. [see http://www.climatescience.gov/Library/stratplan2003/default.htm]
3. National Academy of Sciences (NAS) (2003) *Planning Climate and Global Change Research: A Review of the Draft U.S. Climate Change Science Program Strategic Plan.* Committee to Review the U.S. Climate Change Science Program Strategic Plan, T.E. Graedel, Chair, National Academies Press, Washington, D.C., xiv+85 pp. [see http://www.nap.edu/books/0309088658/html/]
4. Kennedy, D. (2003) The policy drought on climate change. *Science*, **299**, 309.
5. Pielke, R., Jr. and Sarewitz, D. (2003) Wanted: Scientific leadership on climate. *Issues Sci. Technol.* **19**, 27–30.

**This article should be referenced as follows:**

Hanson, H.P. (2003) Down from the ivory tower: Climate science faces the real world. *TheScientificWorldJOURNAL* **3**, 354–356.
